# Two Phase Non-Rigid Multi-Modal Image Registration Using Weber Local Descriptor-Based Similarity Metrics and Normalized Mutual Information

**DOI:** 10.3390/s130607599

**Published:** 2013-06-13

**Authors:** Feng Yang, Mingyue Ding, Xuming Zhang, Yi Wu, Jiani Hu

**Affiliations:** 1 College of Life Science and Technology, Key Laboratory of Image Processing and Intelligent Control of Education Ministry of China, Huazhong University of Science and Technology, Wuhan 430074, China; E-Mails: fyang@foxmail.com (F.Y.); myding@mail.hust.edu.cn (M.D.); wuyihust@hust.edu.cn (Y.W.); 2 School of Computer and Electronics and Information, Guangxi University, Nanning 530004, China; 3 Department of Radiology, Wayne State University, 3990 John R., Detroit, MI 48201, USA; E-Mail: jianihu@yahoo.com

**Keywords:** non-rigid multi-modal registration, Weber local descriptor, sum of squared differences, structural similarity, mutual information

## Abstract

Non-rigid multi-modal image registration plays an important role in medical image processing and analysis. Existing image registration methods based on similarity metrics such as mutual information (MI) and sum of squared differences (SSD) cannot achieve either high registration accuracy or high registration efficiency. To address this problem, we propose a novel two phase non-rigid multi-modal image registration method by combining Weber local descriptor (WLD) based similarity metrics with the normalized mutual information (NMI) using the diffeomorphic free-form deformation (FFD) model. The first phase aims at recovering the large deformation component using the WLD based non-local SSD (wldNSSD) or weighted structural similarity (wldWSSIM). Based on the output of the former phase, the second phase is focused on getting accurate transformation parameters related to the small deformation using the NMI. Extensive experiments on T1, T2 and PD weighted MR images demonstrate that the proposed wldNSSD-NMI or wldWSSIM-NMI method outperforms the registration methods based on the NMI, the conditional mutual information (CMI), the SSD on entropy images (ESSD) and the ESSD-NMI in terms of registration accuracy and computation efficiency.

## 1. Introduction

Non-rigid image registration is one of the most challenging problems in medical image processing. Given two medical images, the objective of the registration process is to find a reasonable non-rigid transformation, such that a transformed version of the float image is similar to the reference one. Despite phenomenal progress in medical image resolution, one modality is often not sufficient to produce a precise diagnosis since different imaging modalities differ in interpreting the anatomy, tissue and organ that they may capture, so multi-modal medical image registration is useful for relating clinically significant information from different images. For example, it can be used to improve the diagnostic tasks and image-guided interventions. However, accurate non-rigid multi-modal registration is highly challenging because of intensity variations and non-rigid transformations between images.

In general, image registration involves three main components: deformation model, similarity metric and optimization strategy. In the non-rigid image registration, the deformation model can be divided into two main categories [1]. The first category is originated from physical models of materials including elastic body models [2], fluid flow models [3] and diffusion models [4]. Another category is relevant to interpolation and approximation theory including radial basis functions [5], elastic body splines [6], free-form deformations (FFD) [7,8].

As regards optimization strategy, numerous optimization methods have been proposed to optimize the parameters of the deformation model in the non-rigid image registration. Examples include gradient descent, Newton's method, Powell's method and discrete optimization [9,10]. Especially, the Broyden–Fletcher–Goldfarb–Shanno (BFGS) algorithm [11] has been used to handle the large number of variables and constraints of registration and is included in the Insight Segmentation and Registration Toolkit (ITK) [12–14].

Apart from deformation model and optimization strategy, similarity metrics have received much attention in the field of image registration. Many similarity metrics have been proposed for different applications [15]. The well-known metrics such as sum of squared differences (SSD) and sum of absolute difference (SAD) have been successfully used for mono-modal image registration, but they are not appropriate for direct application to multi-modal image registration because it is required that the image intensities at corresponding points of two images should be similar [16]. To address this problem, the generalized divergence measure based on Renyi Entropy [17], Kullback-Leibler Divergence [18], mutual information (MI) and cross-cumulative residual entropy (CCRE) [19] have been proposed. Among these metrics, MI has been investigated in-depth and widely applied to multi-modal image registration. MI was firstly introduced to realize the rigid registration of multi-modal scans by Maes *et al.* [20] and Viola *et al* [21]. Rueckert *et al.* [7] extended this similarity metric to non-rigid image registration. Studholme *et al.* proposed the normalized mutual information (NMI) as an overlap invariant generalization of mutual information [22]. Conditional mutual information (CMI) was introduced for non-rigid multi-modal image registration by Loeckx *et al.* to reduce the negative influence of bias fields [23]. However, the traditional global MI approach involves such disadvantages as high computational complexity, tendency to get trapped in local minima and suffering from erroneous transformations of anatomical images even though the MI between the fixed image and the float image achieves the maximum value with this transform [24,25].

In addition to information theory measures-based registration methods, structural representation methods have been investigated for multi-modal medical image registration. Pluim *et al.* [26] utilized the local gradient orientation and Nigris *et al.* [27] used gradient orientations of minimal uncertainty for image registration. In [28], Liu *et al.* proposed a registration method based on the local frequency estimated by calculating the local phase gradient of the most significant Gabor filter response. This method has the advantage that the local frequency is the same for corresponding structures in the two images, even when edge strength and contrast have significant differences. However, the above three methods were used for rigid registration, and not discussed for the non-rigid registration in [26–28]. Heinrich *et al.* [29] utilized modality independent neighbourhood descriptor (MIND) which is not a scalar representation but a vector-valued image descriptor for multi-modal deformable registration. Wachinger *et al.* proposed two structural representation methods. One method utilized the entropy of an image patch to assign a new intensity value and used the SSD on entropy images (ESSD) as similarity metric. Another method firstly used Laplacian Eigenmaps to embed image patches in a lower-dimensional manifold that preserves local distances and then computed L2 distance of Laplacian images. However, the entropy image based method can only meet certain requirements of a relaxed version of the theoretical properties, and the Laplacian image based method involves high computational complexity [30].

To achieve both accuracy and efficiency of the non-rigid multi-modal registration method, a similarity metric based on the Weber local descriptor (WLD) proposed in [31] is combined with the NMI to realize a two phase image registration in this paper. The introduction of the WLD in this paper results from the fact that it can extract image features more effectively than the well-known scale-invariant feature transform and local binary patterns. In the proposed method, the first phase aims at obtaining the parameters (*i.e.*, the control points) relevant to the large deformation, ensuring that the anatomical features of the reference and float images are not destroyed, and reducing the opportunities of getting trapped in local minima by integrating the WLD with the non-local SSD or the structural similarity (SSIM) proposed in [32]. The second phase is focused on getting the parameters related to the small deformation using the NMI to obtain high registration accuracy.

This paper is structured as follows: Section 2 presents the novel two phase non-rigid multi-modal image registration method. Section 3 provides discussions of key parameters in the proposed method and comparisons of registration accuracy and efficiency among our method and the NMI, CMI, ESSD and ESSD-NMI methods. Finally, the conclusion is given in Section 4.

## 2. Methods

### 2.1. Registration Framework

In general, image registration is stated as the following minimization problem:

(1) $$\hat{T}=\mathrm{argmin}f(T;I_{R},I_{F})$$

where *f* (*T*; *I_R_*, *I_F_*) denotes an objective function defined by the similarity metric, and *T* denotes the transformation which is defined as coordinate mapping from the domain of the reference image *I_R_* to that of the float image *I_F_*. To obtain the optimal transformation *T̂*, an appropriate optimization method should be employed.

In Equation (1), it is highly challenging to tackle large deformations. On the one hand, coarse-to-fine deformation schemes have been commonly applied. Wu *et al.* [33] used a wavelet-based deformation model to treat global and local information in a coarse-to-fine approach. The combination of coarse-scale landmark and B-splines deformable registration techniques was proposed in [34]. A hybrid method that combined surface and volume information to register cortical structures was proposed in [35]. Postelnicu *et al.* [36] started with a geometric registration which was used as the initialization and refined it with a non-linear optical flow registration method. On the other hand, to prevent from folding artifacts and preserve local orientation in case of large deformations, the deformation field should be diffeomorphisms. Rueckert *et al.* [8] enforced the transformation of FFD model to be a diffeomorphism by limiting the control points displacement.

**Figure 1.**
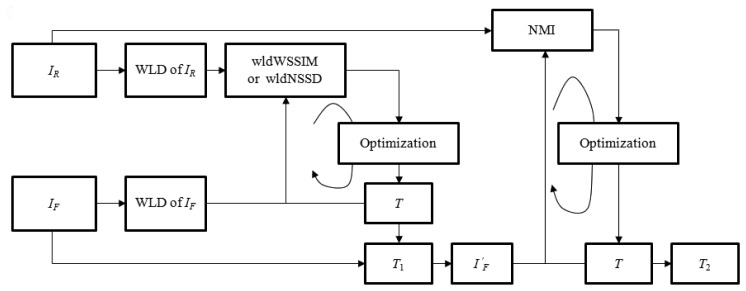
Two phase non-rigid multi-modal image registration.

Different from above-mentioned coarse-to-fine deformation registration methods, the proposed two phase image registration method aims at resolving the above minimization problem by using different similarity metrics in two registration phases as shown in Figure 1. In the large deformation phase, the structural representations of *I_R_* and *I_F_* are firstly obtained using the WLD to compute similarity metric wldNSSD or wldWSSIM. Then, the iterative optimization of the objective function *f*_1_ defined by the wldNSSD or the wldWSSIM is realized to obtain a relatively good initial transformation *T*_1_ while reducing the opportunities of getting trapped in the local minima. However, only using the wldNSSD or the wldWSSIM cannot ensure accurate registration results especially for medical images with complicated deformations because some useful image information may be lost when using the WLD to extract image features. Therefore, a refined registration is implemented by using the original image intensities. In the small deformation phase, the float image *I_F_* is firstly deformed to generate the image $I_{F}^{\prime }$ using *T*_1_ which can provide a good initial value, and then the small deformation for $I_{F}^{\prime }$ is processed for minimizing the objective function *f*_2_ defined by the NMI to obtain the final transformation *T*_2_.

In our method, we used the diffeomorphic FFD model as the deformation model which uses a multi-resolution way by concatenating the FFDs with different grid sizes and limiting the control points displacement less than 0.4 × the grid sizes [8]. Meanwhile, we selected the BFGS algorithm as the optimization method because it is particularly suited to the registration problem with very large numbers of variables without requiring explicit computations.

### 2.2. Large Deformation Phase

#### 2.2.1. Weber Local Descriptor

Ernst Weber observed that the ratio of the increment threshold to the background intensity is a constant in human perception [37]. This relationship between the physical magnitudes of stimuli and the perceived intensity of the stimuli was known as Weber's Law which can be expressed as:

(2) $$\frac{\Delta I}{I}=k$$

where Δ*I* represents the increment threshold, *I* denotes the initial stimulus intensity and *k* signifies that the proportion on the left side of Equation (2) remains constant despite the variation of *I*.

Although Weber's Law describes fundamental relationships of the human perception, *i.e.*, in a biological setting rather than for digital images, several researchers have extended its application to signal and image processing. Dabeer and Onkar introduced a regularized Weber sampler for smooth deterministic signals [38]. Bruni *et al.* used Weber's Law for scratch detection on digital film materials [39]. By using Weber's Law, Chen proposed a Weber local descriptor (WLD) which was used to extract local features [31].

**Figure 2.**
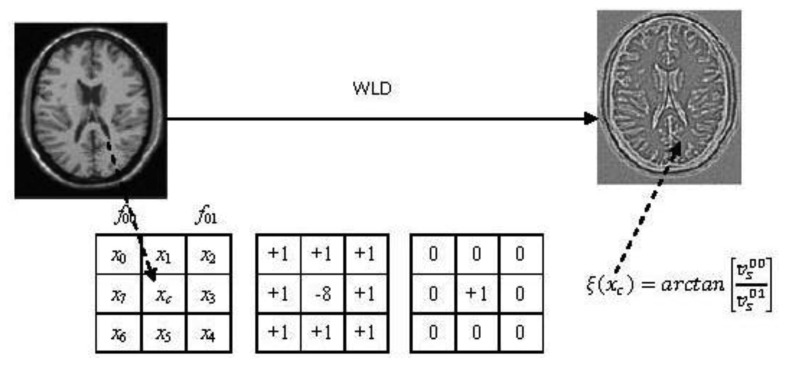
Differential excitation of Weber local descriptor.

The WLD has two components: differential excitation (*ξ*) and orientation (*θ*). In our paper, only differential excitation is used for registration because using both differential excitation and orientation result in increased computational complexity, and differential excitation has such advantages as detecting edges elegantly, robustness to noise and illumination change, and its powerful representation ability for textures [31]. Specifically, a differential excitation *ξ*(*x_c_*)of a current pixel *x_c_* is computed as illustrated in Figure 2. The symbols $v_{s}^{00}$ and $v_{s}^{01}$ are the outputs of the filters *f*_00_ and *f*_01_. It is easy to understand that $v_{s}^{01}=x_{c}$. The difference $v_{s}^{00}$ between the center point *x_c_* and its neighbors is given by:

(3) $$v_{s}^{00}=\sum_{i=0}^{p-1}\left(\Delta x_{i}\right)=\sum_{i=0}^{p-1}\left(x_{i}-x_{c}\right)$$

where *x_i_* (*i* = 0,1,···, *p*-1) denotes the *i*-th neighbors of *x_c_* and *p* is the number of neighbors. The difference $v_{s}^{00}$ is a discrete representation of the Laplace operator. The constancy of Laplacian images is a well-known assumption and has been used e.g., in [40] in the context of optical flow. Normally, this feature is used for invariance under directional changes.

By applying the arctangent function which can limit the output to prevent from increasing or decreasing too quickly when the input becomes larger or smaller [31], the differential excitation *ξ*(*x_c_*) of the pixel *x_c_* is computed as:

(4) $$\xi (x_{c})=\arctan\;\left[\frac{v_{s}^{00}}{v_{s}^{01}}\right]=\arctan\;\left[\sum_{i=0}^{p-1}\left(\frac{x_{i}-x_{c}}{x_{c}}\right)\right]$$

From Equation (4), we can see that although the WLD is not invariant under global brightness changes, it is robust to changes in image contrast. The reason lies in the fact that a change in image contrast in which each pixel value is multiplied by a constant will multiply differences by the same constant, and this contrast change is canceled by the division between $v_{s}^{00}$ and $v_{s}^{01}$ [31]. Here it should be noted that to avoid dividing by zero in Equation (4), a small constant is added to the denominator in practical implementation.

To tailor the WLD to our non-rigid multi-modal registration method, it is expressed as:

(5) $${\text{WLD}}_{R}(I)=\{\xi \;(x_{c},I,R)|x_{c}\;\text{in}\;I\}$$

where *R* denotes the radius of the square symmetric neighborhood.

**Figure 3.**
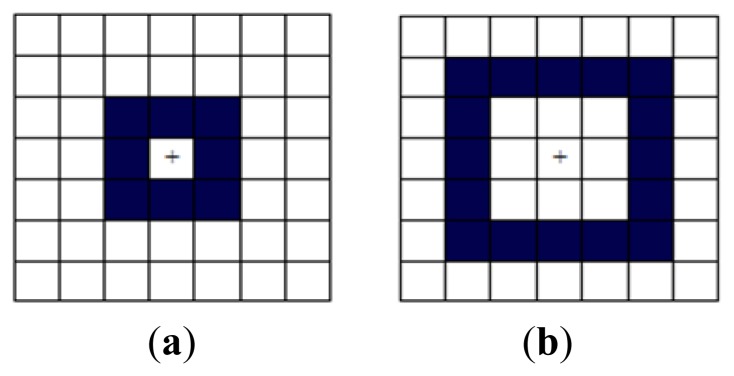
The square symmetric neighborhoods with different *R* for extracting WLD features. (**a**) The neighborhood with *R* = 1. (**b**) The neighborhood with *R* = 2.

Actually, WLD features can be extracted from a square symmetric neighborhood of size (2*R* + 1) × (2*R* + 1). Figure 3 shows examples of the neighborhood with *R* = 1 and *R* = 2. It should be noted that for extracting WLD features, only the border pixels (highlighted in blue in Figure 3) are used instead of all the pixels in the square neighborhood to reduce computation time.

**Figure 4.**
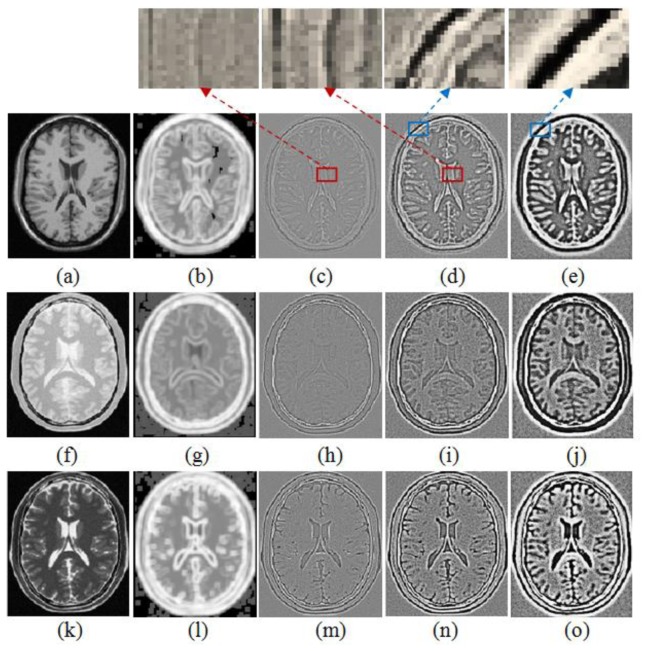
Entropy image and WLD with different *R* of the various MR images. (**a**) deformed T1 image; (**b**) Entropy image of (a); (**c**) WLD with *R* = 1 of (**a**); (**d**) WLD with *R* = 2 of (a); (**e**) WLD with *R* = 3 of (a); (**f**) deformed PD image; (**g**) Entropy image of (f); (**h**) WLD with *R* = 1 of (f); (**i**) WLD with *R* = 2 of (f); (**j**) WLD with *R* = 3 of (f); (**k**) original T2 image; (**l**) Entropy image of (k); (**m**) WLD with *R* = 1 of (k); (**n**) WLD with *R* = 2 of (k); (**o**) WLD with *R* = 3 of (k).

To demonstrate the performance of the WLD with different *R* and its advantage over the entropy image for structural representation, the WLD and the entropy image for deformed T1, PD and original T2 weighted MR images are shown in Figure 4. The comparison between Figure 4(b) and Figure 4(c–e) shows that the detail information in the entropy image of T1 weighted MR images is blurrier than that in the WLD, which indicates that the WLD can provide better structural representation than the entropy image for registration. It should be noted that there exist the black squares and dots in Figure 4(b) because the considered pixel is set to be zero when the minimum intensity is equal to the maximum one in the image patch centered at this pixel. Meanwhile, it is easy to understand that the performance of the WLD changes with different *R*.

From Figure 4(c–e), (h–j) and (m–o), we can see that the WLD with a small *R* (e.g., *R* = 1) generates relatively weak but thin edges and thus facilitates the accurate localization of relatively strong edges. By comparison, the WLD with a larger *R* (e.g., *R* = 2) produces thicker but stronger edges and thus assists with detecting the weak edges while a much larger *R* (e.g., *R* = 3) results in poor edge localization. The zooming of features marked with the red square in Figure 4(c–d) and those marked with the blue square in Figure 4(d–e) can further illustrate the above characteristics. Obviously, the combination of multiple selected *R* for the WLD produces “multi-granuality” features especially in regions with rich textures and discontinuities. Therefore, using the various radii in the WLD is preferable to using a single radius for the effective structural representation of medical images with complicated features. Based on the above analysis, we use the WLD with *R* = 1 and *R* = 2 for structural representation of images.

#### 2.2.2. Weber Local Descriptor Based Non-Local Sum of Squared Differences

To describe the difference between the extracted WLD feature of *I_R_* and that of *I_F_*, the traditional SSD is improved to resist the disadvantageous influence of noise in the medical images. Inspired by the idea of non-local means proposed by Buades *et al.* [41], which aims at image denoising, that the pixel similarity can be represented more effectively using image patches than using individual pixels, the non-local SSD (NSSD) is introduced to represent the difference between the WLD features. The NSSD between two images *I_A_* and *I_B_* is presented as:

(6) $$\text{NSSD}(I_{A},I_{B})=\frac{{\sum_{i=1}^{N}\left\|P_{A}(x_{i})-P_{B}(x_{i})\right\|}_{2}^{2}}{S_{p1}\cdot N}$$

where *N* is the image size, ‖⋅‖_2_ denotes the Euclidean norm, *P_A_*(*x_i_*) and *P_B_*(*x_i_*) mean the square patch of size *S_P_*_1_ centered at *x_i_* of images *I_A_* and *I_B_*, respectively.

As discussed in Section 2.2.1, by combining the WLD using the neighborhoods of different radii *R*_1_ = 1 and *R*_2_ = 2 with the NSSD, we can obtain the following similarity metric wldNSSD:

(7) $$\text{wldNSSD}(I_{R},I_{F})=\text{NSSD}\;\left(\frac{{\text{WLD}}_{R1}(I_{R})+{\text{WLD}}_{R2}(I_{R})}{2},\frac{{\text{WLD}}_{R1}(I_{F})+{\text{WLD}}_{R2}(I_{F})}{2}\right)$$

Based on the wldNSSD, we define the objective function *f*_1_ as:

(8) $$f_{1}(T;I_{R},I_{F})=\text{wldNSSD}(T;I_{R},I_{F})+\gamma C_{\text{smooth}}(T)$$

where *C_smooth_*(*T*) is a regularization term to constrain the FFD transformation to be smooth, γ is the weighting parameter which defines the tradeoff between the alignment of the two images and the smoothness of the transformation. The regularization term takes the following form:

(9) $$C_{\text{smooth}}(T)=\frac{1}{N}\sum_{i=1}^{N}\sum_{j=1}^{2}{\left(\frac{\partial^{2}T_{j}}{\partial x^{2}}(x_{i})\right)}^{2}+{\left(\frac{\partial^{2}T_{j}}{\partial xy}(x_{i})\right)}^{2}+{\left(\frac{\partial^{2}T_{j}}{\partial y^{2}}(x_{i})\right)}^{2}$$

where *N* denotes the number of pixels in *I_R_* or *I_F_*.

#### 2.2.3. Weber Local Descriptor Based Weighted Structural Similarity

Given two images *I_A_* and *I_B_* to be compared, the SSIM involves such components as a luminance (mean) distortion term *l*(*I_A_*, *I_B_*), a contrast (variance) distortion term *c*(*I_A_*, *I_B_*) and a correlation term *s*(*I_A_*, *I_B_*). By combining the three comparisons, the resultant SSIM index between *I_A_* and *I_B_* is presented in a simplified form as [32]:

(10) $$\begin{array}{ll} \text{SSIM}(I_{A},I_{B}) & =l(I_{A},I_{B})\cdot c(I_{A},I_{B})\cdot s(I_{A},I_{B})\\ & =\frac{2{\bar{I}}_{A}.{\bar{I}}_{B}+c_{1}}{{\bar{I}}_{A}^{2}+{\bar{I}}_{B}^{2}+c_{1}}\cdot \frac{2s_{I_{A}}\cdot s_{I_{B}}+c_{2}}{s_{I_{A}}^{2}+s_{I_{B}}^{2}+c_{2}}\cdot \frac{s_{I_{A}.I_{B}}+c_{3}}{s_{I_{A}}.s_{I_{B}}+c_{3}} \end{array}$$

where *C*_1_,*C*_2_ and *C*_3_ are small constants for characterizing the saturation effects of the visual system at the regions of low luminance and contrast and ensuring numerical stability when the denominators are close to zero; ${\bar{I}}_{A},{\bar{I}}_{B},s_{I_{A}}^{2},s_{I_{B}}^{2},$ and *S_I_A__,_I_B__* represent the local mean of *I_A_* and *I_B_*, the local variance of *I_A_* and *I_B_*, and the local covariance between *I_A_* and *I_B_*, respectively. The first two terms *l*(*I_A_*, *I_B_*) and *c*(*I_A_*, *I_B_*) account for nonstructural distortion of the image, whereas the last term accounts for structural distortion of the image.

The SSIM index is not a metric. However, the distance $D=\sqrt{1-c(I_{A},I_{B})\cdot s(I_{A},I_{B})}$ is a single scalar-valued distance measure [42]. In [32], a mean SSIM has been used for image quality assessment. However, it is helpful to improve the performance of image quality assessment algorithms by giving different weights to different image patches [43]. Therefore, the WLD difference based weighted SSIM (wldWSSIM) is adopted as a metric:

(11) $$\text{WSSIM}(I_{A},I_{B})=\sqrt{1-\frac{\sum_{j=1}^{N}w\left(P_{A}(x_{j}),P_{B}(x_{j})\right)\cdot c\left(P_{A}(x_{j}),P_{B}(x_{j})\right)\cdot s\left(P_{A}(x_{j}),P_{B}(x_{j})\right)}{\sum_{j=1}^{N}w\left(P_{A}(x_{j}),P_{B}(x_{j})\right)}}$$

where *P_A_*(*x_j_*) and *P_B_*(*x_j_*) mean the square patch of size *S_P_*_2_ centered at *x_j_* of images *I_A_* and *I_B_*, *N* is the image size and the weight *w*(*P_A_*(*x_j_*), *P_B_*(*x_j_*)) is computed as:

(12) $$w\left(P_{A}(x_{j}),P_{B}(x_{j})\right)=\frac{1}{1+{\left\|P_{A}(x_{j})-P_{B}(x_{j})\right\|}_{2}}$$

Similar to the wldNSSD, the similarity metric wldWSSIM is defined as:

(13) $$\text{wldWSSIM}(I_{R},I_{F})=\text{WSSIM}\;\left(\frac{{\text{WLD}}_{R1}(I_{R})+{\text{WLD}}_{R2}(I_{R})}{2},\frac{{\text{WLD}}_{R1}(I_{F})+{\text{WLD}}_{R2}(I_{F})}{2}\right)$$

Based on the wldWSSIM, the objective function *f*_1_ is defined as:

(14) $$f_{1}(T;I_{R},I_{F})=\text{wldWSSIM}(T;I_{R},I_{F})+\gamma C_{\text{smooth}}(T)$$

where the regularization term *C_smooth_*(*T*) and the constant γ are given as in Equation (8).

#### 2.2.4. Analysis of the wldNSSD and the wldWSSIM

**Figure 5.**
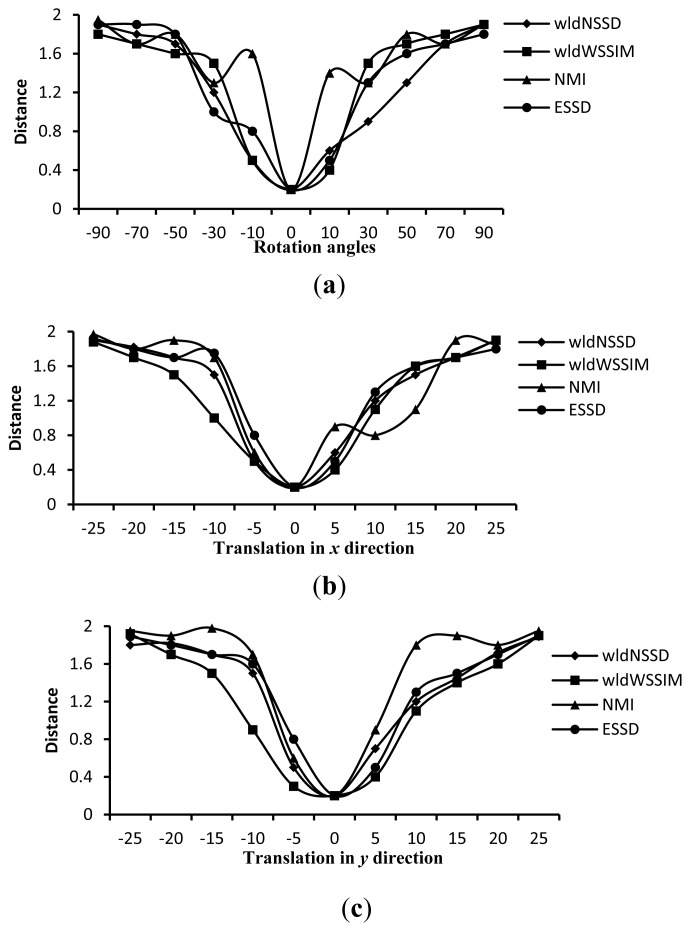
Distance measures for T1 and T2 weighted MR images. (**a**) Distance versus rotation angles; (**b**) Distance versus translation in *x* direction (mm); (**c**) Distance versus translation in *y* direction (mm).

To demonstrate the advantage of the wldNSSD and the wldWSSIM over such metrics as the NMI and the ESSD, we compute the four distance measures on ten pairs of T1 and T2 weighted MR images rotated around the domain center with different angles and translated in *x* and *y* directions. Figure 5 shows an excerpt of the corresponding computation results. We can see from Figure 5 that the wldNSSD and wldWSSIM have no local extremes while the NMI has local extremes in Figure 5(a–c), and the ESSD involves local extremes in Figure 5(b). Similar findings also exist for other computation results, which indicate that the wldNSSD and the wldWSSIM are more effective similarity metrics for registration than the NMI and the ESSD.

### 2.3. Small Deformation Phase

To obtain more accurate registration results, the small deformation phase is needed for the refined registration. In this phase, the float image *I_F_* is firstly deformed using *T*_1_ which is the output of the large deformation phase. So, we have $I_{F}^{\prime }=T_{1}\;(I_{F})$. The small deformation is processed by minimizing the objective function *f*_2_ defined by the NMI between $I_{F}^{\prime }$ and *I_R_*:

(15) $$f_{2}(T;I_{R},I_{F}^{\prime })=\text{NMI}(T;I_{R},I_{F}^{\prime })+\gamma C_{\text{smooth}}(T)$$

where *C_smooth_*(*T*) and γ are given as in Equation (8), and the NMI is defined as:

(16) $$\text{NMI}(I_{R},I_{F}^{\prime })=\frac{H(I_{R})+H(I_{F}^{\prime })}{H(I_{R},I_{F}^{\prime })}$$

where *H* denotes the Shannon entropy. $H(I_{R},I_{F}^{\prime })$, *H* (*I_R_*) and $H(I_{F}^{\prime })$ are defined as:

(17) $$H(I_{R},I_{F}^{\prime })=-\sum_{f\in B_{F}}\sum_{r\in B_{R}}p(r,f)\log P(r,f)$$

(18) $$H(I_{R})=-\sum_{r\in B_{R}}p_{R}(r)\log p_{R}(r)$$

(19) $$H(I_{F}^{\prime })=-\sum_{f\in B_{F}}p_{F}(f)\log p_{F}(f)$$

where *B_R_* and *B_F_* are sets of regularly spaced intensity bin centres, *p* is the discrete joint probability, 𝑝*_R_* and 𝑝*_F_* are the marginal discrete probabilities of the reference image *I_R_* and the float image $I_{F}^{\prime }$, respectively [44].

## 3. Experiments

In this section, to determine the key parameters in the proposed method and make comparisons of registration performance among our method, the NMI method, the CMI method, the ESSD method and the ESSD-NMI method, extensive experiments have been performed on thirty T1, T2 and PD-weighted MR images of size 256 × 212 pixels from the BrainWeb database [45]. Registration efficiency of all these evaluated methods is appreciated by their computation time (in seconds) when implemented in a multi-resolution way by rescaling the FFD grid spacing 2*^k^* × 2*^k^* (each image is rescaled to a square of size 2 to the *k*th power) [8] using MATLAB 2010 on a personal computer with 2.40 GHz CPU and 4 GB RAM. Registration accuracy is appreciated by target registration error (TRE) [46] with simulated deformation and expert landmark annotations as ground truth, respectively.

As regards the simulated deformation *T_s_*, it is used as the ground truth to deform one of two different weighted MR images (e.g., T1 and T2). By implementing the various registration methods on the float image and the generated reference image, the estimated deformation *T_c_* will be obtained. Based on the whole image domain, TRE*_d_* is computed as:

(20) $${\text{TRE}}_{d}=\frac{{\left\|T_{s}(I)-T_{c}(I)\right\|}_{2}}{\left|I\right|}$$

where |*I*| denotes the size of the whole image domain *I*.

When we use expert landmark annotations as ground truth, for a estimated deformation *T_c_* and a set of anatomical landmark pairs $\left\{I_{LF},I_{LR}\}=\{(x_{i},y_{i}),(x_{i}^{\prime },y_{i}^{\prime })\right\}$ (*i*=1, 2,···, *m*, where *m* is the number of anatomical landmarks), TRE*_l_* is defined as:

(21) $${\text{TRE}}_{l}=\frac{{\left\|T_{c}(I_{LF})-I_{LR}\;\right\|}_{2}}{m}$$

**Figure 6.**
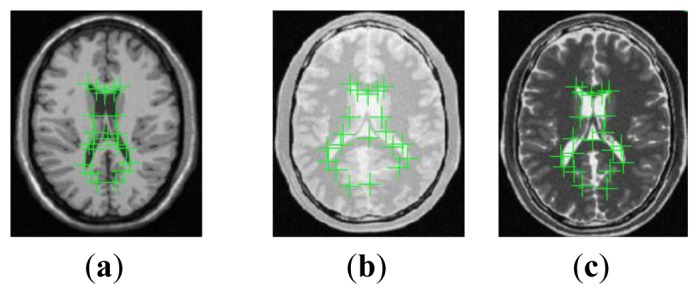
Landmarks in the deformed T1, deformed PD and original T2 images. (**a**) Landmarks in the Deformed T1; (**b**) Landmarks in the Deformed PD; (**c**) Landmarks in the original T2.

Considering that expert landmark annotations based evaluation requires the anatomical landmarks in the reference image *I_R_* and the float image *I_F_* to be marked, we have invited five experts to manually select twenty landmarks defined according to the anatomical structures including the left and right lateral ventricles for the deformed T1, PD and original T2 images shown in Figure 6.

### 3.1. Choice of Parameters

#### 3.1.1. Choice of the patch size

The patch sizes *S_P_*_1_ and *S_P_*_2_ are very important for the similarity metrics wldNSSD and wldWSSIM. To quantitatively determine these parameters, we discuss their influence on registration accuracy and efficiency by using fifteen images.

**Figure 7.**
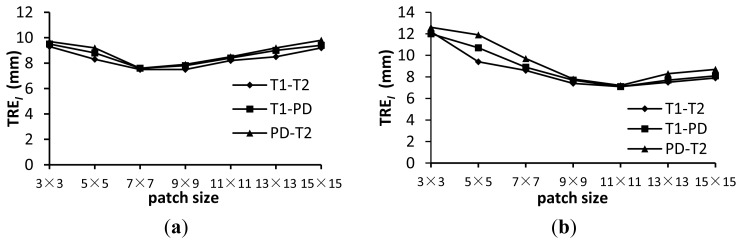
TRE*_l_* (in mm) for the wldNSSD and wldWSSIM using various patch sizes. (**a**) wldNSSD; (**b**) wldWSSIM.

**Figure 8.**
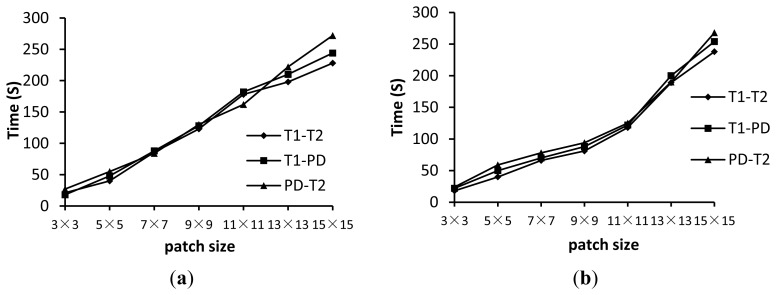
Time (in seconds) for the wldNSSD and wldWSSIM using various patch sizes. (**a**) wldNSSD; (**b**) wldWSSIM.

Figure 7 shows the TRE*_l_* for the wldNSSD and wldWSSIM methods using various patch sizes. Figure 8 shows the computation time for the two metrics using various patch sizes. We can see from Figure 7 that a too small or too large patch size has a disadvantageous influence on registration accuracy. For the wldNSSD method, the TRE*_l_* can achieve the minimum value of 7.5 mm when the patch size is 7 × 7 or 9 × 9 while the optimal patch size is 11 × 11 for the wldWSSIM method. Meanwhile, it is shown in Figure 8 that the computation time increases with the increasing patch size because a larger patch size means that more pixels need to be processed. Therefore, to achieve the tradeoff between registration precision and efficiency, the patch size is chosen to be 7 × 7 for the wldNSSD method and 11 × 11 for the wldWSSIM method in our experiments. It should be noted that the wldWSSIM method needs bigger patches for higher registration performance than the wldNSSD. The reason can be explained in this way. The wldNSSD depends on the difference of intensities of two WLD feature images while the wldSSIM takes into account not only the local mean and the local variance, but also the local covariance of two WLD feature images for representing their structural similarity. Therefore, for the wldWSSIM method, a larger patch size is needed to ensure the statistical significance.

#### 3.1.2. Choice of the Weighting Term γ

The weighting term γ is specific to the processed images. To quantitatively determine γ, we make some tests on γ with fifteen T1, T2 and PD-weighted MR images.

**Figure 9.**
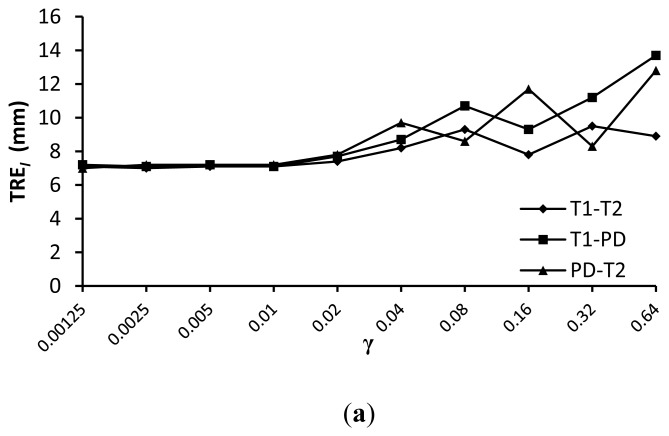

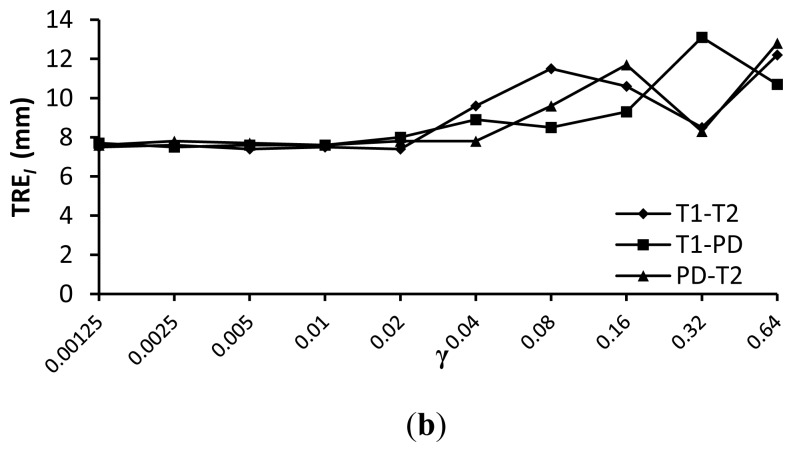
Comparison of registration precision with different γ for wldWSSIM and wldNSSD. (**a**) wldWSSIM; (**b**)wldNSSD.

The comparison results of registration precision with different γ for similarity measures wldWSSIM and wldNSSD are shown in Figure 9. We can see from this figure that a value of γ =0.01 provides good registration results for the various weighted MR images. The reason lies in two aspects. On the one hand, the target registration error TRE*_l_* becomes unstable with a larger γ because it will reduce the impact of the similarity measure which is important for registration precision. On the other hand, the regularization term which is used to constrain the FFD transformation to be smooth is less important because we have limited the control point displacement less than 0.4 × the grid sizes in the FFD model.

### 3.2. Comparison of Registration Performance

To demonstrate the advantage of the wldNSSD and wldWSSIM, wldNSSD-NMI and wldWSSIM-NMI methods, they are compared with other evaluated methods in terms of registration accuracy and efficiency.

The mean and standard deviation (std) of TRE*_d_* and TRE*_l_* for all the evaluated methods are shown in Table 1 and Table 2. Here, “/” in Tables 1 and 2 means that no registration is implemented. Obviously, the wldNSSD and wldWSSIM methods can achieve a smaller TRE*_d_* and TRE*_l_* than the NMI, CMI and ESSD methods. Meanwhile, we can see that the wldNSSD-NMI and wldWSSIM-NMI methods for two deformation phases can achieve higher registration accuracy than the corresponding wldWSSIM and wldNSSD methods, which demonstrates the advantage of combining the WLD similarity metrics with the NMI. It should be noted that the NMI method can be seen as a two phase method since it was implemented in a multi-resolution way in our test, but this method achieves a bigger TRE*_d_* and TRE*_l_* than the wldWSSIM-NMI and wldNSSD-NMI methods because the NMI method is easy to get trapped into local minima only using the image intensity information. Besides, the intra-observer errors and inter-observer errors are listed in Table 2 for reference. It is shown that the smallest registration error for the wldWSSIM-NMI method is 6.1 mm and it is still higher than the inter-observer errors. The main reason is that experts were requested to repeat the marking procedure if necessary to ensure that the inter-observer errors are less than 5.0 mm in the case of the large simulated deformation among different weighted MR images.

Table 3 lists the computation time of all these methods. The observation from Table 3 shows that our methods have less computation time than the NMI, CMI, ESSD and ESSD-NMI methods and thus they outperform these compared methods in terms of registration efficiency. Moreover, the wldWSSIM-NMI method is of lower registration efficiency than the wldNSSD-NMI while the former can provide higher registration accuracy. Therefore, our two phase methods can be applied according to the clinical requirements. If one application attaches more significance to registration accuracy, the wldWSSIM-NMI method is better choice. If registration efficiency is more important, the wldNSSD-NMI method is preferable.

**Figure 10.**
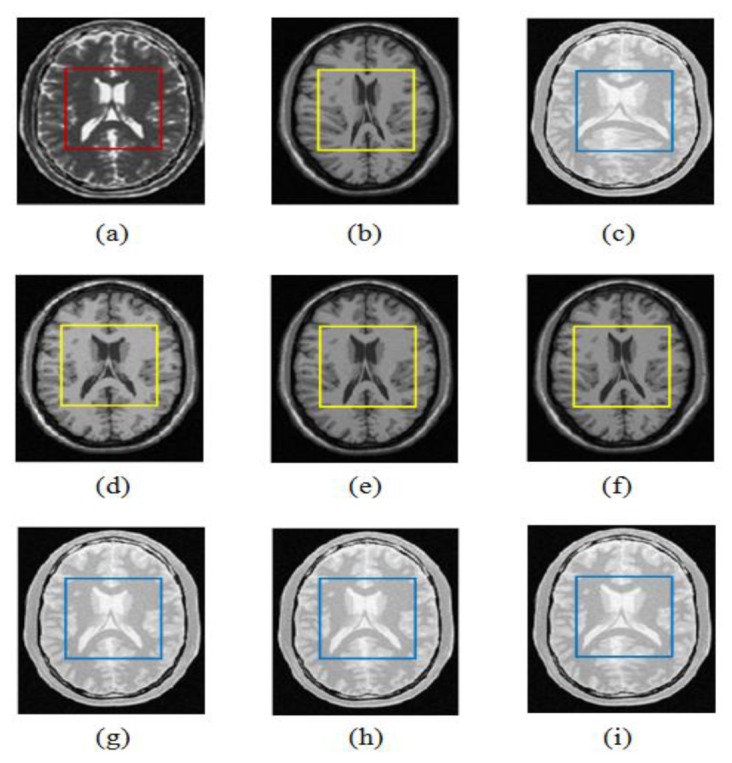
Non-rigid multi-modal registration results for the wldWSSIM-NMI, wldNSSD-NMI and ESSD-NMI methods operating on T1-T2 and PD-T2 weighted MR images. (**a**) T2 (the reference image); (**b**) T1 (the float image which is deformed by compressing mainly in the lateral ventricle marked with a yellow square.); (**c**) PD (the float image which is deformed by expanding mainly in the lateral ventricle marked with a blue square.); (**d**) wldWSSIM-NMI (T1-T2); (**e**) wldNSSD-NMI (T1-T2); (**f**) ESSD-NMI (T1-T2); (**g**) wldWSSIM-NMI (PD-T2); (**h**)wldNSSD-NMI (PD-T2); (**i**) ESSD-NMI (PD-T2).

Figure 10 shows an example of T1-T2 and PD-T2 weighted MR images and registration results for the wldWSSIM-NMI, wldNSSD-NMI and ESSD-NMI methods. From Figure 10(d–f), we can see that the results using the wldWSSIM-NMI and wldNSSD-NMI methods are anatomically more similar to the reference T2 weighted MR image than those using the ESSD-NMI, especially in the lateral ventricle which is marked with a yellow square. Similar results can be seen in Figure 10(g–i).

## 4. Conclusions

In this paper, we have proposed a two phase non-rigid multi-modal medical image registration method using the Weber local descriptor based similarity metrics and the normalized mutual information. In the first phase, the parameters relevant to the large deformation are obtained by minimizing the objective function defined by the novel similarity metric wldNSSD or wldWSSIM which is focused not on intensities of individual pixels but on structural information of images. With the good initial deformation value provided by the output of the large deformation phase, the parameters related to the small deformation can be accurately obtained using the NMI in the second phase. The non-rigid image registration experiments on the T1, T2 and PD weighted MR images demonstrate that compared with the NMI, CMI, ESSD and ESSD-NMI methods, our method can obtain smaller registration errors and higher computational efficiency. Future work will be focused on extending our method to multi-modal 3D medical image registration.

## Figures and Tables

**Table 1.** TRE*_d_* (in mm) for all the evaluated methods.

**Similarity Metric**	**TRE*_d_* (mm)**					

	**T1-T2**		**T1-PD**		**PD-T2**	
		
	**mean**	**std**	**mean**	**std**	**mean**	**std**
**/**	2.8	2.2	3.0	2.4	2.9	2.4
**NMI**	1.5	1.1	1.7	1.2	1.7	1.3
**CMI**	1.4	1.0	1.5	1.2	1.6	1.2
**ESSD**	1.4	0.9	1.6	1.1	1.5	1.1
**ESSD-NMI**	1.4	0.9	1.5	1.1	1.5	1.1
**wldNSSD**	1.2	0.7	1.3	0.8	1.3	0.9
**wldNSSD-NMI**	0.9	0.5	1.0	0.7	1.0	0.6
**wldWSSIM**	1.0	0.6	1.1	0.7	1.1	0.7
**wldWSSIM-NMI**	0.8	0.4	0.9	0.5	0.9	0.5

**Table 2.** TRE*_l_* (in mm) for all the evaluated methods.

**Similarity Metric**	**TRE***_l_* **(mm)**					

	**T1-T2**		**T1-PD**		**PD-T2**	
		
	**mean**	**std**	**mean**	**std**	**mean**	**std**
**Intra-observer errors**	0.9	0.7	1.0	0.8	1.0	0.8
**Inter-observer errors**	4.9	4.2	5.0	4.4	4.9	4.2
**/**	15.4	9.2	15.7	9.3	15.6	9.5
**NMI**	9.5	6.2	9.8	6.3	9.6	6.8
**CMI**	8.9	5.8	8.6	5.6	9.1	6.2
**ESSD**	8.6	5.7	8.6	5.6	8.9	6.2
**ESSD-NMI**	8.4	5.6	8.4	5.6	8.7	5.8
**wldNSSD**	7.4	4.9	7.7	5.0	7.6	5.0
**wldNSSD-NMI**	6.7	3.8	6.8	3.9	6.8	3.9
**wldWSSIM**	7.1	4.0	7.1	4.1	7.2	4.2
**wldWSSIM-NMI**	6.1	3.3	6.2	3.4	6.2	3.5

**Table 3.** Computation time (in seconds) for all the evaluated methods.

**Similarity Metric**	**Time (s)**		

	**T1-T2**	**T1-PD**	**PD-T2**
**NMI**	248 ± 20	268 ± 22	280 ± 31
**CMI**	350 ± 28	346 ± 26	366 ± 26
**ESSD**	224 ± 16	230 ± 15	246 ± 16
**ESSD-NMI**	284 ± 24	292 ± 21	294 ± 28
**wldNSSD**	86 ± 7	88 ± 9	84 ± 9
**wldNSSD-NMI**	119 ± 12	118 ± 12	123 ± 14
**wldWSSIM**	118 ± 11	122 ± 10	125 ± 11
**wldWSSIM-NMI**	148 ± 14	152 ± 15	157 ± 15
